# Structural and Practical Departments

**Published:** 1906-07-07

**Authors:** 


### STRUCTURAL AND PRACTICAL DEPARTMENTS.

### HOSPITAL BEDSTEADS.

(Messrs. Monks, Hall and Company, Limited, Standard Bedstead Works, Warrington.)

The "Monkshall" bedstead has most, if not all, of the latest improvements in hospital bedsteads. One defect, however, which could be easily rectified is that the bed is too low. Here we have the mattress about 18 inches from the floor, whereas, especially for medical bedsteads, it is essential it should be at least 2 feet or 2 feet 3 inches from the floor, so as to necessitate as little stooping as possible on the part of the physicians. The bars at the head of the bed are arranged so as to prevent bolster or pillows falling through, and every attention has been paid to reducing the amount of ornamentation, so as to make the surface as smooth as possible. The castors are good, and there is an excellent arrangement for fitting wooden feet when castors are objected to. The steel-wire mattresses are clean and well constructed, and have all the appearance of being of good wearing material. The arrangement for moving the head of the bedstead for the administration of anæsthetics is a simple one—namely, pieces of iron at each end sliding down the tubes which form the back legs. There is really no better or more simple method than this; almost every type where hinges are employed are complicated, and likely to get out of order. The best feature of the bedstead is the construction of the steel-tube sides, which are perfectly smooth and strong, and well fitting to head and foot pieces, thus constituting a very excellent hospital bed. In addition the price works out at less than most similar bedsteads now in the market.

